# A New and Important Therapeutic Property of Quinine

**Published:** 1871-11

**Authors:** 


### A New and Important Therapeutic Property of Quinine.

The following conclusions are arrived at in a memoir of 277 pages, by Doctor Monteverdi.

1. The action of quinine and its preparations is especially upon the grand sympathetic nervous system.

2. This action causes contraction of the muscular fibres supplied by the sympathetic, particularly those of the uterus, urinary bladder, the intestinal canal, and the blood-vessels.

3. This action may be physiological according to the amount of quinine given. If the fibres are relaxed, quinine restores their tone, and enables them to perform their functions. If they are contracted and excited, quinine produces a rigid tonic contraction, which renders them unable to perform their functions.

4. This property is especially marked on pregnant women, on whom quinine must be used with care, as it is able to and frequently has caused premature labor.

5. In labor it may be often used with certainty and advantage to stimulate the uterine contractions, for which it is preferable to ergot for the following reasons: It does not injure mother or fœtus; it acts very rapidly and surely; it produces regular, intermittent labor pains, precisely normal in character; it may be administered at any stage of the labor, from before the bag of waters is ruptured or the os dilated, to the time when the head is on the perineum.

6. In cases of the retention of the placenta, either after labor or miscarriage, it is of great value, causing expulsion, and replacing manual assistance.

7. In menorrhagia in pregnant or confined women, it is to be preferred to ergot, as safer and more certain.

8. In amenorrhœa from uterine inertia, it is of great service.

9. In puerperal fever it is very useful, not only as a preventative, but also to combat victoriously the disease in its early stages.

10. In hysteria and hysteralgia it increases the uterine excitement, and more often is hurtful than beneficial.

11. In all the diseases of the digestive apparatus, or of the genito-urinary system, it is useful when they depend upon atony; if irritation or inflammation be present, it does harm. In the normal condition of the bladder it will sometimes cause such a rigid, persistent contraction of the neck, as to render the evacuation of urine difficult, or even impossible.

12. Small doses of quinine augment the force and frequency of the heart's action; large doses enfeeble it.

13. Opium and morphia are antagonistic to quinine, and are useful when it has produced toxic symptoms.

14. To produce physiological effects upon the uterus, to excite contraction, etc., 20–25 centigrammes (3 to 4 grains) should be given at each dose; to produce the paralytic effect, the dose should exceed a gramme (15.43 grs.).—*Annales et Bulletin de la Societe de Medecine de Gand*, May, 1871.—*New Remedies.*

